# Safety and efficacy of the treatment of chronic anal fissure by lateral internal sphincterotomy: A retrospective cohort study

**DOI:** 10.1016/j.amsu.2020.08.010

**Published:** 2020-08-12

**Authors:** Fatma Al-thoubaity

**Affiliations:** Department of Surgery, King Abdulaziz University Hospital, Jeddah, Saudi Arabia

**Keywords:** Chronic anal fissure, Lateral internal sphincterotomy, Recurrence, Incontinence, Patient satisfaction

## Abstract

**Background:**

An anal fissure is one of the most common anorectal diseases resulting from a longitudinal tear in anoderm under the dentate line. It causes severe pain during defecation, and the resulting emotional stress leads to a reduction in the overall quality of life of a person. There are several medical and surgical treatment procedures that are employed to treat anal fissures. In the present retrospective study, we aimed to evaluate the safety and efficacy of lateral internal sphincterotomy (LIS) surgical procedure for the treatment of chronic anal fissure. Additionally, we also analyzed the complications arising after the surgery and the factors affecting the complications.

**Methods:**

The present study is a retrospective study that included 691 patients treated for chronic anal fissure in a private sector setting, by a single Saudi female surgeon, starting from April 2004 to April 2020.

**Results:**

Out of the 691 patients included in the study, all the patients were female, with an average age of 45.77 years (ranging from 17 to 82 years). Major complaints of the patients were as follows: pain, bleeding, constipation, pruritus, perianal discharge. Recurrence was reported in 2 patients (0.3%) while, 3 patients (0.4%) developed gas incontinence.The complaints of all patients with gas incontinence regressed over a period of time.

**Conclusion:**

This study reports that lateral internal sphincterotomy (LIS) is a safe surgical treatment for chronic anal fissure, leading to a reduction in recurrence, incontinence rate and substantially improves symptoms, especially pain.

## Highlights

• In patients with chronic anal fissures, the majority of the patients had a posterior anal fissure.
• Lateral internal anal sphincterotomy (LIS) is a safe and effective surgical treatment for chronic anal fissure.
• LIS procedure results in high patient satisfaction rates and improvement in the quality of life.
• Incontinence and recurrence rate are very low in our study.

## 1 Introduction

An anal fissure is a common anorectal problem. It is a painful longitudinal tear in the anoderm distal to the dentate line [1]. The patients are usually diagnosed upon presenting the symptoms such as significant pain during defecation with a variable amount of rectal bleeding [2]. Along with the physical pain, this condition can result in tremendous emotional stress, which causes an overall decline in the quality of life of a person [3]. Usually, 90% of the anal fissures are located in the posterior midline, while anteriorly located fissures are less common (10%), and found more frequently in women [1,4]. While the acute anal fissure often heals within 1–2 weeks, chronic anal fissures are less likely to heal even after 6–8 weeks of medical management [5,6]. Treatment of anal fissure is focused on reducing the pressure of internal sphincter muscle with the help of physical or chemical methods [3]. The treatment strategy of chronic anal fissures varies from conservative medical management to surgery [[1], [2], [3]].

Conservative methods of medical management are used first to heal an anal fissure. Medical therapies, such as the application of local anaesthetic, fiber diet, and topical nitroglycerin aid in the early healing of fissure [2]. Similarly, The American Society of Colon and Rectal Surgeons (ASCRS) guidelines recommend that for the initial nonsurgical management of anal fissure, the patient should be recommended stool softeners, high fiber diet, and warm sitz bath [7]. Application of pharmacological agents such as glyceryl trinitrate or calcium blockers, and botulinum toxin (BT) injection are other treatment strategies, which are also termed as “chemical sphincterotomy” [[8], [9], [10], [11]]. However, the success rate (65–75%) of this treatment strategy is significantly lower than as observed in surgical sphincterotomy [[11], [12], [13]].

Since the last 3-4 decades, lateral internal sphincterotomy (LIS) has been the preferred method for treating chronic anal fissures [14]. With a success rate as high as 96%–100%, LIS is considered the gold standard for the surgical management of chronic anal fissures, when the conservative medical treatments fail [3,6,15]. However, in around 3% of the patients, there are instances of wound-related complications such as bleeding, abscess or non-healing wound, and fistula [16,17]. The other major feared complications associated with LIS are recurrence of anal fissure and incontinence [6]. The results have shown that the recurrence rate is usually below 10%; however, high anal incontinence rates up to 30% have been observed in some studies [4,18]. However, in most cases, incontinence is transient, and most of the patients recover after postoperative management [19].

The primary objective of this study is to understand the safety and effectiveness of the lateral internal anal sphincterotomy (LIS) for the treatment of chronic anal fissure in a cohort of patients. Preoperative complaints of the patients were recorded and were further analyzed for subsequent weeks, postoperatively, for the relief in the pain due to anal fissure. Further, we also analyzed the early and late complications arising from the LIS; recurrence of anal fissures and incontinence in the patients were among the major complications we studied. Finally, patient satisfaction, as a measure of improvement in the overall health quality, was recorded post LIS. Overall, this study enhances our knowledge about the importance of LIS, as a procedure, in treating chronic anal fissures.

## 2 Methods

### 2.1 Ethical considerations

There were minimal ethical implications and issues since it is a retrospective study. Patient identity and confidentiality were protected by assigning each patient with a specific serial number. Prior approval was obtained from the Institutional Review Board at the Bakhsh Hospital; Ethical Approval number- 29102019. Since it is a retrospective study, the consent of the patient was not required for the study. The work has been reported in line with the STROCSS criteria [20].

### 2.2 Study design

The present study is a retrospective study, where 691 female patients were included who underwent surgery for the treatment of chronic anal fissure. Lateral internal sphincterotomy (LIC) procedure was performed on the patients by the open method in the private sector Bakhsh Hospital in Jeddah, Saudi Arabia, from April 2004 to April 2020. All patients in the study were operated upon by the author previously. Before the surgery, medical management for all the patients was done, which included the prescription of a combination of stool softener, laxative, high fiber diet, and a warm sitz bath.

Inclusion criteria for the patients included in the present study were as follows: a female patient suffering from anal fissure for more than six weeks, exposed internal anal sphincter fibers, the appearance of sentinel piles, and hypertrophied anal papilla. Patients who were suffering from an anal abscess, anal fistulae, hemorrhoid disease, and inflammatory bowel disease were excluded from the study. Most patients had a history of normal delivery, and no patient had any history of anal or rectal cancer.

Lateral internal sphincterotomy (LIS) surgical procedures were performed with the open method in a lithotomy position. The procedures were performed as a daycare procedure under general or regional anesthesia. Prophylactic antibiotics and rectal enemas were not used. The anal canal was visualized with the help of an anoscope. A longitudinal incision of about 1 cm was made, at 3 o clock, in the intersphincter groove and an artery was used to separate the muscle from the mucosa. The distal half of the internal anal sphincter, below dentate line, was divided under direct vision followed by application of pressure for 3 min, and then wound was closed using vicryl 3-0 suture and a small dressing was applied at end of procedure. Marcaine (0.25% 10 cc) was injected locally to relieve postoperative pain, while Voltarene Suppositories (100 mg) was administered into the rectum to decrease the postoperative pain.

### 2.3 Postoperative management and follow up

Wound dressing (Kaltostat) was removed after 6 h of the surgery. Paracetamol (1 g) intravenous every 6 h, and a narcotic (Pethidine 50 mg IM q6h) if needed. Oral metronidazole was administered, 500 mg twice daily, for one week postoperative. NSAID (non-steroidal anti-inflammatory drug) (Voltarene 50 mg p.o. q8h), stool softeners (Agiolax, one spoon po daily), local xylocaine gel 2% every 6h, and mebo ointment applied locally to the wound (q6h), were also prescribed to the patient at the time of hospital discharge.

Anorectal examination of the patients was performed in every follow-up clinic visit, and the fissure healing was monitored; the postoperative follow-up visits were scheduled after 1 week, then after 2 weeks, 4 weeks, and 8 weeks, respectively. Pain relief was assessed using a visual analog scale representing the severity of pain from 0 (no pain) to 10 (worst imaginable pain).

The medical records of the patients were analyzed retrospectively. The parameters that were analyzed included patient's age, medical history, symptoms and findings, and response to the treatment (pain relief and evaluation of the fissure, erythema, and inflammation). Subsequently, the side effects of the treatment and incidences of the disease's recurrence were also recorded and analyzed.

## 3 Results

Table 1 showed the clinical profile of the patients included in this study. Of the 691 patients included in the present study, all the patients were female, and the mean age of the patients was 45.77 ± 8.96 years (ranging from 17 to 82 years) (Table 1). In the majority of the patients, the primary complaints of the patients were pain during defecation (97.4%) and rectal bleeding (77.9%) during or after defecation (Table 1). Additionally, the other significant complaints of the patients were constipation (98.0%), pruritis (17.0%), and perineal discharged (4.0%) (Table 1). In the majority of the cases, the anal fissure was posteriorly located (67.0%), while the remaining patients had anteriorly located anal fissure (33.0%) (Table 1).

**Table 1:** Clinical profile of the study group.

Clinical features	*n* (%)
Gender	
Female	691 (100%)
Age (years); Mean ± SD	45.77 ± 8.96
**Complaints**	
Pain during defecation	673 (97.4%)
Rectal bleeding	538 (77.9%)
History of constipation	677 (98.0%)
Pruritus	118 (17.0%)
Perianal discharge	28 (4.0%)
Anterior anal fissure	228 (33.0%)
Posterior anal fissure	463 (67.0%)

The patients were evaluated for the relief in pain after the first, second, fourth, and eighth week following surgery. The patients’ response towards the treatment and the status of their complaints was assessed (Table 2). In the first week, 435 patients (63.1%) observed significant improvement in their pain, and subsequently, 532 patients (77.2%) in the second week, 601 patients (87.0%) in the fourth week and 636 patients (92.0%) in the eighth week observed relief in pain (Table 2). It is also important to note that 55 patients did not observe any relief in pain, even at the end of the eighth week (Table 2).

**Table 2:** Results of pain relief.

Number of patients whose complaints relieved	*n* (%)
First week	435 (63.1%)
Second week	532 (77.2%)
Fourth week	601 (87.0%)
Eighth week	636 (92.0%)

The median duration of the disease was 27 months (ranging from 1 to 34 months). Table 3 shows the early and late complication outcomes of the procedure, recorded in the follow-up during the study (16 years). In the early postoperative period, rectal bleeding was not observed to be a common problem because only 5 patients (0.7%) were affected by it (Table 3). Also, only 2 patients (0.3%) had a perineal abscess, and one patient (0.2%) had a perineal hematoma, and that patient used anticoagulants; these patients were relieved of the problems after the drainage of the abscess and control of bleeding from a hematoma (Table 3).

**Table 3:** Early and late complications outcome reported during 16 year follow up.

Complications	*n* (%)
Rectal bleeding	5 (0.7%)
Perianal abscess	2 (0.3%)
Perianal hematoma	1 (0.2%)
Recurrence	2 (0.3%)
Incontinence	3 (0.4%)
Patient satisfaction	670 (97.0%)
Fissure healing	677 (98.0%)

In the long term follow up, recurrence of anal fissure occurred in 2 patients (0.3%), and 3 patients (0.4%) developed incontinence for gas, which improved with time, and regressed in the fourth postoperative month (Table 3). The patients who had a recurrence also had a history of previous surgery. The female with incontinence was elderly, and one patient had multiple vaginal deliveries.

Patient satisfaction was found to be high (93.0%), and the healing was nearly complete (98.0%) at the end of the eight weeks.

## 4 Discussion

An anal fissure is a common disease of the anal canal, which leads to a vicious cycle of pain, sphincter spasm, and inflammation [6]. Our study's primary aim was to determine the safety and efficacy of lateral internal anal sphincterotomy (LIS) to treat chronic anal fissure. Further, particular emphasis was laid on analyzing significant complications associated with the LIS, such as recurrence and incontinence. Additionally, overall patient satisfaction and the healing of the anal fissure is reported in the study.

There are different strategies to manage and treat chronic anal fissure. In the initial phase, some studies recommend conservative and medical treatment strategies as they are non-invasive and avoid the risk of anal sphincter injury [2,21]. An earlier study observed that dietary bran supplements and warm sitz baths effectively treat acute anal fissures [22]. The other medical management process is termed as “chemical sphincterotomy”, where pharmacological agents such as glyceryl trinitrate or calcium blockers, and botulinum toxin (BT) injection are applied to treat chronic anal fissures [[8], [9], [10], [11]]. However, it is not considered an effective treatment strategy and has a low success rate [6,23]. Therefore, lateral internal sphincterotomy is the most preferred treatment due to its high rate of success and is also considered gold-standard for surgical management of chronic anal fissures [2,3,12].

Our study group included 691 female patients (no male), who suffered from chronic anal fissures. During the initial investigations, it was found that a large number of patients (98.0%) had a history of constipation (Table 1). The patients complained of pain during defecation (97.4%), rectal bleeding (77.9%) and pruritus (17.0%), and these observations are similar to the ones where patients suffering from anal fissures complained of pain (96–100%), rectal bleeding (80%) and pruritus (39%) [6,[24], [25], [26]]. The perianal discharge was reported by fewer patients (4%) as compared to other complaints. It is also worth noting that most patients (67%) had a posterior anal fissure, while significantly fewer patients had an anterior anal fissure (Table 1). These observations are in concurrence with the earlier studies wherein the majority of the patients had anal fissures located on the posterior midline [2,3,6].

Next, pain relief in the patients was measured postoperatively. The number of patients whose pain got relieved improved consistently from the first week of the surgery (63.1%) to the second week (77.2%), fourth week (87.0%), and at the end of the eighth week, most patients (92.0%) experienced pain relief (Table 2). These results were similar to a previous study of Arujo et al., where maximum pain relief was observed at the end of the eighth week [27]. In another study, it was observed that almost all the patients had pain relief after six weeks of the surgery [28].

Although LIS procedure is highly successful, there are instances of different complications during the early stages as well as the late stages post-surgery. The early complications that were in the present study were rectal bleeding (0.7%), perianal abscess (0.3%), and perianal hematoma (0.2%) (Table 3). Similar early complications have been reported in earlier studies [6].

Other complications arising from LIS are anal incontinence, which is a major disadvantage of LIS [2]. It is estimated that up to 50% of the patients suffer from transient incontinence, which includes the inability to control gas, and involuntary loss of feces [2]. In a study analyzing the results of 22 studies, 4512 patients, it was observed that anal incontinence resolves itself in most of the patients, but in less than 2% of the patients, major incontinence (involuntary loss of feces) was observed [2,29]. In line with the previous studies, we observed that only 0.4%, patients showed gas incontinence, which itself got improved after some time.

While success rates of the LIS procedure remain high, as judged by the successful healing of the anal fissure, there is a risk of recurrence in 1.3%–25% of the cases [30,31]. Liang et al. also obtained similar results, where the recurrence rate was only 4% [6]. In the present study, the recurrence of the anal fissure was seen in only 2 patients, thereby yielding a very low recurrence rate of 0.3% (Table 3). A common reason for the recurrence is inadequate sphincterotomy, and in such instances, sphincterotomy can be repeated. However, there is very little known about the success of repeat LIS on recurrent anal fissure [3,6]. Despite the instances of complications post LIS, the overall satisfaction of the patients in our study was at 97.0%, and in 98.0% of the patients, complete healing of fissures was observed.

## 5 Conclusion

This study reports that lateral internal sphincterotomy (LIS) is a safe and effective surgical treatment for chronic anal fissure, and leads to a reduction in recurrence and substantially improves the pain symptoms. The present study supports the surgical intervention by LIS procedure because of the high percentage of fissure healing and patient satisfaction. Although gas incontinence was observed in a few patients, high-risk patients must be closely monitored by measuring sphincter pressure. Additionally, supportive Botox treatment can be considered for high-risk patients to reduce the chances of incontinence further.

## Provenance and peer review

Not commissioned, externally peer reviewed.

## Funding

The work was not supported or funded by any drug company.

## Ethical Approval

There were minimal ethical implications and issues since it is a retrospective study. Patient identity and confidentiality were protected by assigning each patient with a specific serial number.

Prior approval was obtained from the Institutional Review Board at the Bakhsh Hospital; Ethical Approval number- 29102019.

## Consent

Since it is a retrospective study, the consent of the patient was not required for the study.

## Registration of research studies

1. Name of the registry: Research Registry
2. Unique Identifying number or registration ID: researchregistry5795
3. Hyperlink to your specific registration (must be publicly accessible and will be checked): https://www.researchregistry.com/browse-the-registry#home/

## Guarantor

Fatma Al-thoubaity.

## Disclosure

The author declares no competing financial interest. Furthermore, the work was not supported or funded by any drug company.

## Author contribution

Study concept or design - Fatma Al-thoubaity

Data collection- Fatma Al-thoubaity

Data analysis or interpretation- Fatma Al-thoubaity

Writing the paper- Fatma Al-thoubaity

## Declaration of competing interest

The author declares no competing financial interest.
